# Interrelation of pericoronary adipose tissue texture and coronary artery disease of the left coronary artery in cardiac photon-counting computed tomography

**DOI:** 10.3389/fcvm.2024.1499219

**Published:** 2024-12-05

**Authors:** Jannik Kahmann, Dominik Nörenberg, Theano Papavassiliu, Stefan O. Schoenberg, Matthias F. Froelich, Isabelle Ayx

**Affiliations:** ^1^ Department of Radiology and Nuclear Medicine, University Medical Center Mannheim, Heidelberg University, Mannheim, Germany; ^2^ First Department of Medicine-Cardiology, University Medical Center Mannheim, Heidelberg University, Mannheim, Germany

**Keywords:** photon-counting computed tomography, pericoronary adipose tissue, texture analysis, coronary artery calcification, radiomics

## Abstract

**Aim:**

Recent research highlights the role of pericoronary adipose tissue (PCAT) in coronary artery disease (CAD) development. PCAT has been recognized as a metabolically active tissue involved in local inflammation and oxidative stress, potentially impacting CAD initiation and progression. Radiomics texture analysis shows promising results to better understand the link between PCAT quality and CAD risk. Photon-counting CT (PCCT) offers improved feature stability and holds the potential for advancing radiomics analysis in CAD research.

**Methods:**

In this retrospective, single-center, ethic committee-approved study, PCAT of the left descending artery (LAD) and right coronary artery (RCA) was manually segmented and radiomic features were extracted using pyradiomics. The study population consisted of one group of patients with CAD and plaques exclusively located in the left coronary artery and another group without CAD. Mean and standard deviation were calculated using R Statistics. Random forest feature selection was performed to identify differentiating features between the four sets CAD-LAD, CAD-RCA, non-CAD-LAD and non-CAD-RCA.

**Results:**

36 patients were enrolled in this study (16 female, mean age 56 years). The feature “original_glszm_GrayLevelNonUniformity” measuring the gray-level variability was identified as the most potent differentiator between CAD-LAD and non-CAD-LAD, as well as CAD-RCA and non-CAD-RCA with the greatest differentiating capability for the LAD comparison. The feature showed little differentiating power between CAD-LAD and CAD-RCA and virtually none between non-CAD-LAD and non-CAD-RCA. The mean values were consistently lower in LAD-PCAT and exhibited patient-specific reductions in CAD patients (155.16 for CAD-LAD, 163.21 for non-CAD-LAD, 189.13 for CAD-RCA and 215.40 for non-CAD-RCA).

**Conclusion:**

Radiomics analysis revealed differences in PCAT texture of patients with and without CAD with a potentially more homogeneous pattern in CAD-affected patients. These changes related to plaques in the left coronary artery also seemed to occur in the unaffected RCA-PCAT, although to a slightly lesser extent.

## 1 Introduction

CAD remains a leading cause of morbidity and mortality worldwide (1). While traditional risk factors such as hypertension, diabetes, and dyslipidemia play pivotal roles in its pathogenesis, emerging research has highlighted the multifaceted nature of this complex disorder (2). PCAT, the adipose depot surrounding coronary arteries, has garnered increasing attention due to its potential contribution to vascular inflammation and CAD development (3, 4). Recent studies have suggested that the texture of PCAT might serve as a novel and previously underappreciated indicator of CAD risk and risk for major adverse cardiovascular events (MACE) (5).

Pericoronary adipose tissue, once regarded as a mere bystander to coronary artery health, is now recognized as a metabolically active tissue capable of both beneficial and detrimental effects on the vasculature. Conventionally, PCAT was thought to provide mechanical support and insulation to coronary arteries. However, mounting evidence suggests its active participation in local inflammation, oxidative stress, and cytokine production, which can contribute to the initiation and progression of atherosclerosis—the hallmark of CAD (4, 6). The influence of pericoronary adipose tissue texture on the development of coronary artery disease represents a rapidly expanding field of research that holds promise for a deeper understanding of CAD pathophysiology and risk stratification (3, 7).

The endorsement of cardiac computed tomography angiography (CCTA) is already the primary diagnostic choice for patients with low-to-intermediate pre-test probability for coronary artery disease, with comparable diagnostic value and fewer procedure-related complications compared to initial invasive coronary angiography (8, 9). Traditionally, this refers to the subjective assessment of qualitative visual features, which heavily relies on the examiner's expertise. As a result, it may not always fulfill the need for accuracy and inter-examiner comparability that modern medicine requires (10). Now, significant advancements in imaging techniques, particularly in cardiac computed tomography (CT), have enabled detailed evaluation of tissue texture through radiomics analysis (11). Radiomics texture analysis involves the quantification of pixel intensity patterns within an image, thereby providing insights into tissue heterogeneity, composition, and structural attributes (12). In the context of PCAT, texture analysis holds promise in elucidating the intricate interplay between adipose tissue quality and CAD risk. Studies have shown that variations in PCAT texture might be indicative of underlying metabolic changes and inflammation, potentially contributing to CAD pathogenesis (5, 13).

To realize the full potential of radiomics texture analysis, it will be important to overcome certain constraints that currently hinder the integration of radiomics analysis into clinical protocols. Notably, its potentially low reproducibility due to technical parameters, related to reconstruction algorithms, contrast and layer thickness may pose a limitation (14, 15).

The novel PCCT technology, in comparison to traditional energy-integrating detector (EID) based CT devices, employs smaller photon-counting detector (PCD) elements. These PCD elements have the distinct capability of promptly converting incident photons into electrical impulses upon contact with the detector plate (16). This pivotal distinction equips PCCT with enhanced spatial resolution, amplified signal-to-noise ratio, and reduced beam hardening artifacts (16, 17). Exploiting these merits, PCCT demonstrates improved feature stability, presenting a promising avenue for advancing radiomics analysis and surmounting several of the mentioned limitations (18, 19). Additionally, the novel PCCT technology allows a better coronary stenosis assessment of calcified plaques, leading to a lower percentage of diameter stenosis using ultrahigh-spatial resolution technique in comparison to standard resolution as recently outlined by Halfmann et al. (20) The combination of both advantages could improve cardiovascular risk assessment.

Hence, the aim of this study is to investigate whether an alteration in the tissue texture of PCAT surrounding unaffected coronary arteries in patients with CAD is detectable in comparison to healthy individuals without CAD, searching for an imaging biomarker in the early detection of arteriosclerosis.

## 2 Material and methods

### 2.1 Study design

Between April 2022 and July 2022, this retrospective single-center study enrolled patients who presented with a clinical indication for contrast-enhanced cardiac CT as outlined in the European Society of Cardiology (ESC) guidelines (8). Exclusions were made for patients with a prior pacemaker or cardiac stent implantation, as well as those exhibiting severe image artifacts, including motion artifacts. Clinical parameters were extracted retrospectively from a pre-existing questionnaire about traditional clinical risk factors. The study adhered to the principles of the Declaration of Helsinki and received approval from the institutional review board and local ethics committee (ID 2021-659).

### 2.2 Patient cohort

Based on inclusion and exclusion criteria, 18 patients (7 female, 11 male, mean age 57 years, range 40–71 years) with CAD and plaques exclusively in the left coronary artery (LCA) were selected. Out of these 18 patients, 3 patients (17%) suffered from CAD with coronary artery stenosis of at least 50%, whereas the other 15 patients (83%) demonstrated a stenosis degree of less than 50%. The stenosis degree was estimated visually and additionally measured using radial reconstructions by a senior radiologist. Matching 18 patients (9 female, 9 male, mean age 54 years, range 38–72 years) without CAD were selected for comparison purposes. Matching was performed in terms of age, sex and cardiovascular risk factors as far as possible on the PCCT dataset cohort. A detailed overview of the patients’ characteristics is offered in Table 1.

**Table 1:** Patient cohort overview.

Patient characteristics	Overall	Non-CAD	CAD	*p*-value
*n*	36	18	18	
Age	55.55	53.93	57.17	0.33
Sex m/f	20/16	9/9	11/7	0.52
Agatston Score	60.49	0	120.97	0.00
Hypertension (%)	10 (28%)	5 (28%)	5 (28%)	1.00
Hypercholesterolemia (%)	3 (8%)	2 (11%)	1 (6%)	0.56
Diabetes (%)	4 (11%)	2 (11%)	2 (11%)	1.00
Nicotine (%)	11 (31%)	5 (28%)	6 (33%)	0.73

Mean and (SD) given for continuous variables.

### 2.3 Cardiac CT imaging

A total of 36 patients underwent examination using a first-generation whole-body dual-source PCCT system (NAEOTOM Alpha; Siemens Healthcare GmbH, Forchheim, Germany). The examination employed a prospective electrocardiographic (ECG)-gated sequential mode, utilizing a tube voltage of 120 kV and automatic dose modulation. The CARE keV BQ setting was set at 64, and the gantry rotation time was 0.25 s. All examinations were conducted at the same effective kV of 120 kV. To achieve heart rates below 65 beats per minute, patients were administered intravenous β-blockers in the range of 5–10 mg, if not contraindicated and based on individual heart rates. Sublingual nitroglycerin (0.8 ml) was also administered afterward. A non-enhanced cardiac CT scan was conducted initially to assess coronary artery calcification. Subsequently, a contrast-enhanced scan was performed using 80 ml of iodine contrast (Imeron 400, Bracco Imaging Deutschland GmbH, Konstanz, Germany) along with a 20 ml saline chaser (NaCl 0.9%) at a weight-based flow rate of 5–6 ml/sec. The initiation of CCTA was triggered by bolus tracking in the descending thoracic aorta.

### 2.4 Cardiac CT imaging analysis

**Figure 1:**
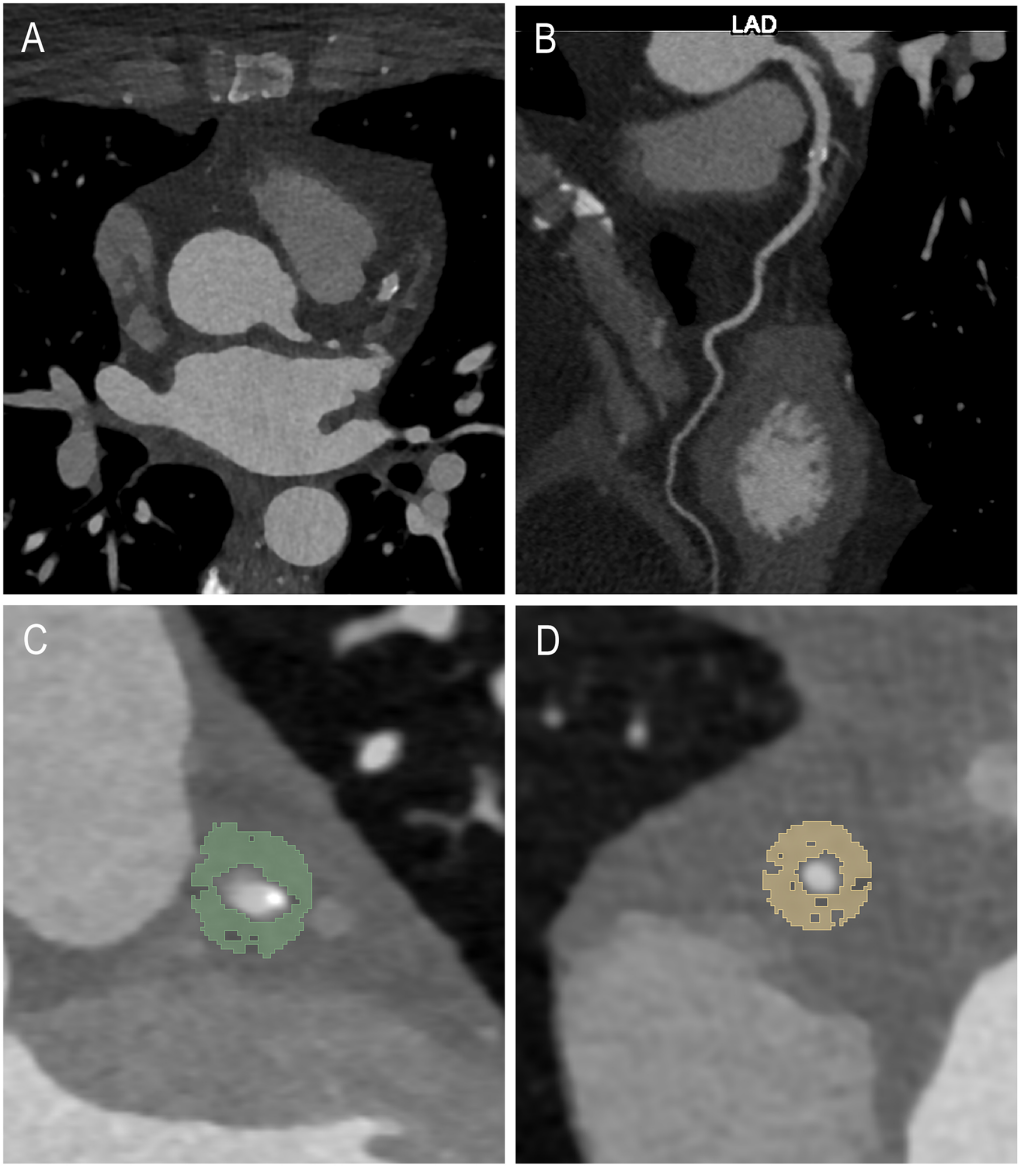
Example case of a 53-year-old female with a plaque in the LAD including the axial view **(A)**, a multiplanar reconstruction of the LAD **(B)** and example segmentations of the LAD **(C)** and RCA **(D)**.

Coronary artery calcification assessment involved non-enhanced axial scans with a 3 mm slice thickness and Qr36 kernel, utilizing dedicated syngo.via software (Siemens Healthcare GmbH, Forchheim, Germany). Axial contrast-enhanced CCTA images were reconstructed using a soft vascular kernel (Bv40), with a slice thickness of 0.6 mm and an increment of 0.4 mm. After anonymization, these images were exported as Digital Imaging and Communications in Medicine (DICOM) files, subsequently converted to Neuroimaging Informatics Technology Initiative (NIFTI) format, and imported into 3D Slicer (Version 4.11), a dedicated segmentation tool (21). Coronary arteries were assessed regarding plaque morphology and degree of stenosis by a senior radiologist with a decade of cardiothoracic imaging experience and six years of segmentation expertise. Pericoronary adipose tissue segmentation of the CAD cohort and the non-CAD comparison cohort was conducted manually for the plaque-affected LAD (CAD-LAD) and the non-plaque affected RCA (CAD-RCA) in the CAD cohort and for the non-pathological LAD (non-CAD LAD) and RCA (non-CAD RCA) in the non-CAD comparison group respectively by a medical student with over one year of segmentation experience, validated by the same experienced senior radiologist. The pericoronary adipose tissue, defined as voxels between −30 and −190 Hounsfield units (HU) surrounding the RCA or LAD, was segmented along a 6 cm path beginning 1 cm past the RCA ostium and along 4 cm starting immediately distal of the bifurcation of the left main artery (LM). Segmentation was performed within a radial distance equal to the underlying vessel's diameter from the outer vessel wall. Figure 1 offers an axial view illustrating an example of pericoronary adipose tissue segmentation.

### 2.5 Radiomics feature extraction and statistical analysis

Radiomics features, including shape, first-order statistics, grey level co-occurrence matrix (glcm), grey level dependence matrix (gldm), grey level size zone matrix (glszm), grey level run length matrix (glrlm) and neighboring grey tone difference matrix (ngtdm), were derived from the LAD and RCA segmentations of the CAD and non-CAD cohorts respectively using pyradiomics (version 3.0.1) (22). The collected features were then imported into R Statistics (version 4.2.0, R Core Team, Vienna, Austria) (23) and subjected to analysis within RStudio (version 2022.07.1 + 554, Boston, MA) (24) for statistical evaluation. Means and standard deviations were calculated. All radiomics features were then normalized by z-score transformation:

z=(X-μ)/σ

Four sets of extracted features were compared against each other: LAD and RCA of CAD patients and LAD and RCA of non-CAD patients. Random forest (RF) analysis was performed with the randomForest package for R to determine the feature with the highest differentiating ability between the groups. The feature best differentiating between the CAD LAD group and the non-CAD LAD group was further assessed regarding its capability of differentiating between the other groups. Correlation plots including regression lines and correlation factors of the comparisons of CAD LAD vs. CAD RCA and non-CAD LAD vs. non-CAD RCA were generated.

## 3 Results

### 3.1 Plaque assessment

Coronary arteries of all patients with CAD were assessed regarding plaque morphology and degree of stenosis. Plaques were categorized as soft, calcified, or mixed, while the degree of stenosis was evaluated as greater or less than 50%. A total of 56 plaques could be identified in the 18 patients. Most of these plaques (50) were calcified, five were soft, and only one was assessed as mixed. Three patients presented with a total of six plaques with a degree of stenosis greater than 50%. Five of these plaques were calcified, and one was soft. Four out of 56 plaques had high-risk-plaque features (7.1%), consisting of two cases of spotty calcifications (3.6%), one case of positive remodeling (1.8%) and one case of napkin ring sign (1.8%). Table 2 offers an overview of these results.

**Table 2:** Plaque characteristics of patients with CAD.

Total (> 50%)	Soft (> 50%)	Calcified (> 50%)	Mixed (> 50%)
56 (6)	5 (1)	50 (5)	1 (0)

Number of plaques and number of plaques with degree of stenosis >50%.

### 3.2 Feature selection

**Figure 2:**
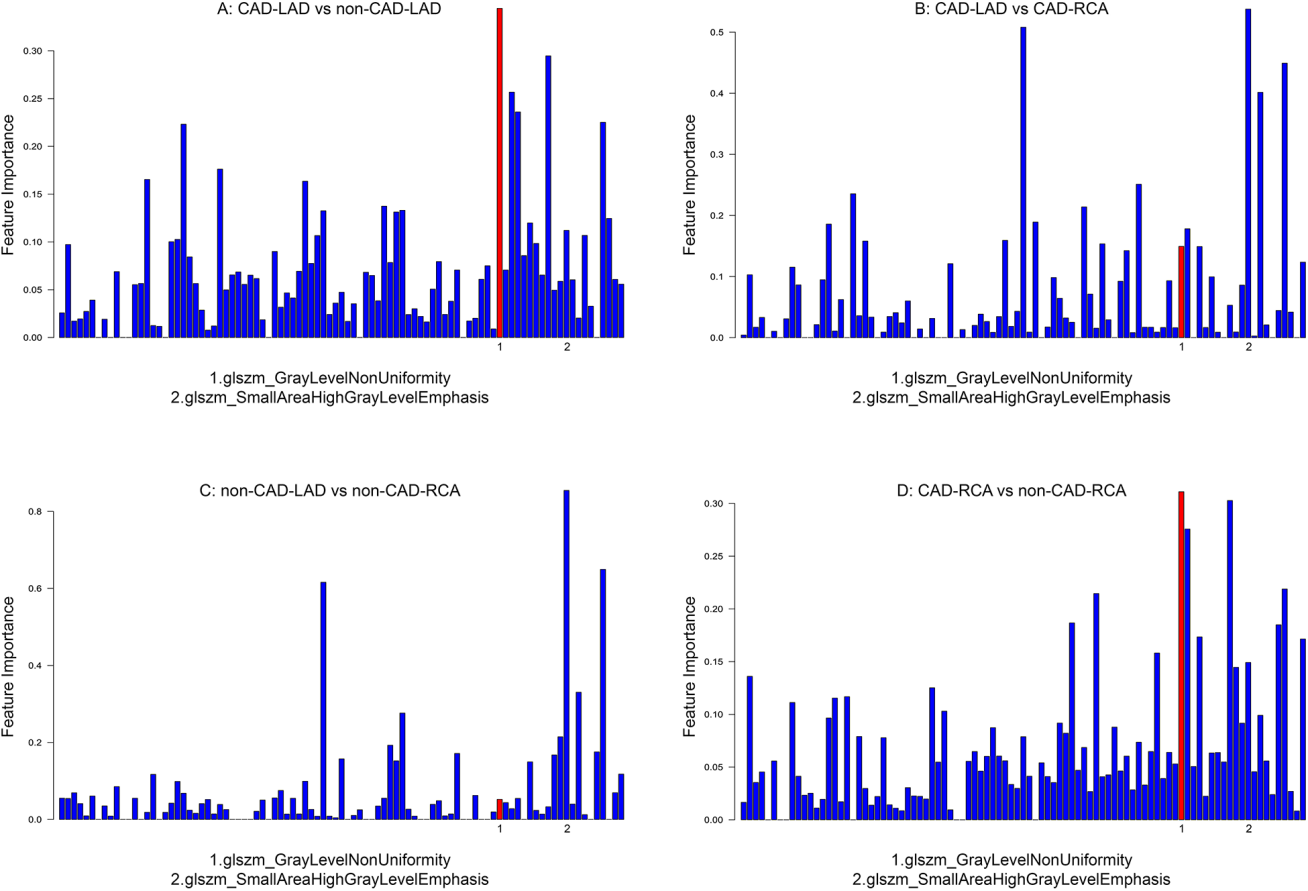
Results of random forest analysis. Highlighted in red is the feature “original_glszm_GrayLevelNonUniformity”, selected for best differentiation between PCAT of CAD and non-CAD patients.

Important features capable of distinguishing the PCAT texture across the four groups were identified using RF analysis. The algorithm was performed for four different comparisons: CAD LAD vs. non-CAD LAD, CAD LAD vs. CAD RCA, CAD RCA vs. non-CAD RCA, and non-CAD LAD vs. non-CAD RCA. The feature with the highest differentiating ability between LAD and RCA PCAT texture was “original_glszm_SmallAreaHighGrayLevelEmphasis” in CAD patients as well as in non-CAD patients. This feature belongs to the Gray Level Size Zone Matrix category which describes the distribution of zones (connected groups of voxels with the same intensity). It measures the relation between small zones and high-intensity voxels with a higher value indicating a texture pattern dominated by many small zones of high intensity (25). The feature best differentiating between the LAD of CAD patients and the LAD of non-CAD patients was “original_gslzm_GrayLevelNonUniformity”. It also showed the best capability of distinguishing the RCA of CAD patients from the RCA of non-CAD patients. This feature also belongs to the Gray Level Size Zone Matrix and calculates the variability of gray levels across all zones. In this case, a higher value indicates a less uniform distribution with certain gray levels appearing notably more frequently than others (25). To improve the readability of the following text, we will refer to this feature as “Gray-Level Variability”. Gray-Level Variability's differentiating ability was assessed in all four groups. This comparison showed a declining differentiating power in the following order: 1. CAD LAD vs. non-CAD LAD, 2. CAD RCA vs. non-CAD RCA, 3. CAD LAD vs. CAD RCA, 4. non-CAD LAD vs. non-CAD RCA (Figure 2).

Gray-Level Variability showed lower mean values in CAD patients, with the LAD having an even lower mean (155.16) than the RCA (189.13). While the mean values for this feature were higher in patients presenting without CAD for each vessel respectively, the mean value of the LAD (163.21) was once again lower in comparison to the RCA (215.40). In summary, the mean value of this feature was consistently lower in LAD-PCAT and exhibited patient-specific reductions in CAD patients (Table 3).

**Table 3:** Original_firstorder_mean and original_glszm_grayLevelNonUniformity for LAD and RCA in patients with and without CAD.

	non-CAD LAD	non-CAD RCA	CAD LAD	CAD RCA
Original_firstorder_Mean	−95.97 (10.31)	−93.00 (11.16)	−99.74 (9.04)	−94.41 (9.65)
Original_glszm_GrayLevelNonUniformity	163.21 (54.97)	215.40 (80.10)	155.16 (45.01)	189.13 (53.65)

Mean and (SD) given for continuous variables.

### 3.3 Correlation plots

**Figure 3:**
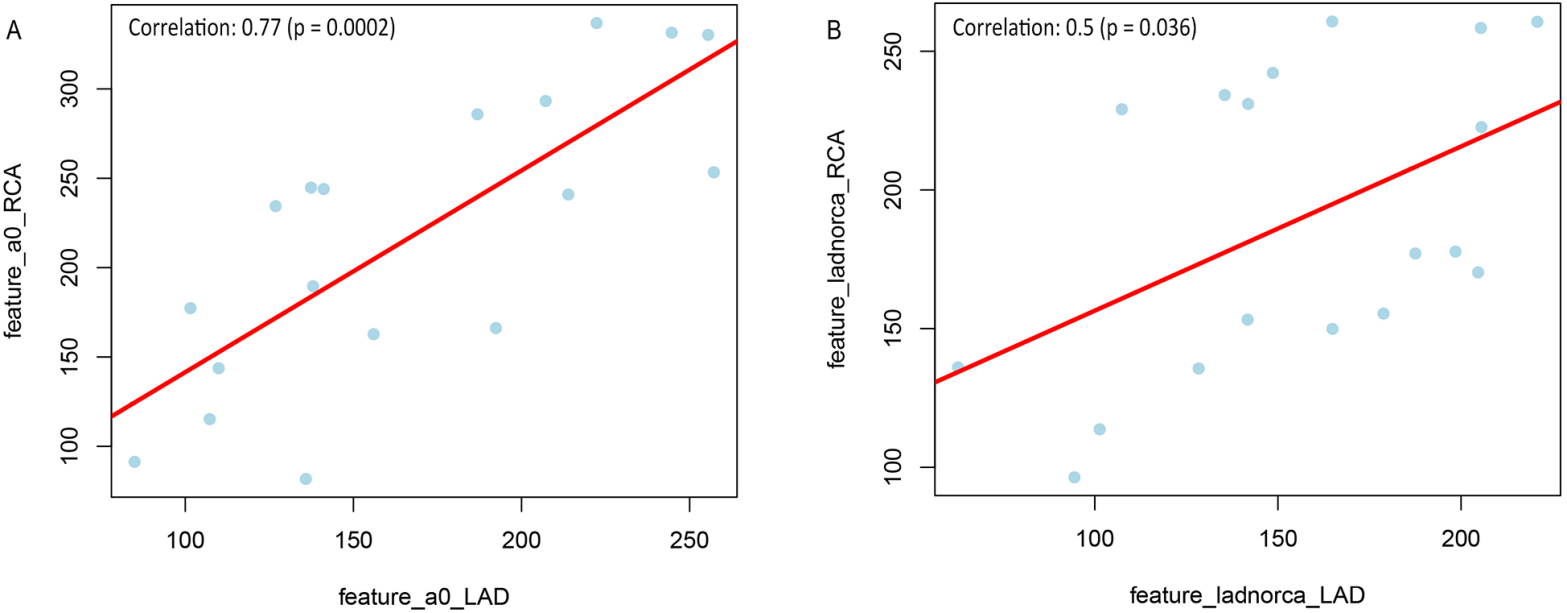
Correlation of the selected feature “original_glszm_grayLevelNonUniformity” in LAD and RCA of patients with and without CAD **(A)** non-CAD LAD vs. non-CAD RCA; **(B)** CAD-LAD vs. CAD-RCA).

Two correlation plots including regression lines and correlation factors were generated to illustrate the correlation of Gray-Level Variability between both arteries within the CAD as well as the non-CAD group (Figure 3). The results showed a higher correlation for the comparison of the two non-CAD arteries (0.77), followed by the comparison of the two CAD arteries (0.5).

## 4 Discussion

In this study, we demonstrated that radiomics feature analysis of PCAT texture may offer a way to discriminate between patients with and without CAD and that plaques exclusively located in the LCA seem to affect PCAT texture in both the left and right coronary arteries of the patient with plaques solely located in the LCA. Not only did Gray-Level Variability differ between the directly affected LAD of CAD patients and the LAD of coronary healthy individuals, but it also showed the highest potential out of all extracted features to differentiate between the only indirectly CAD-affected RCAs and their healthy counterparts. On closer look, RF analysis revealed that the feature showed a gradually declining differentiating power in the following order: 1. CAD LAD vs. non-CAD LAD, 2. CAD RCA vs. non-CAD RCA, 3. CAD LAD vs. non-CAD LAD, 4. non-CAD LAD vs. non-CAD RCA. This indicates that the most important difference exists between the directly CAD-affected LAD and the unaffected healthy LAD, followed by the indirectly affected and the healthy RCA, suggesting that structural changes of LAD-PCAT related to plaques in the LCA also occur in RCA-PCAT, although to a slightly lesser extent. In accordance with these findings, there seems to be a less important difference between the LAD and RCA in CAD patients reflecting the strongest effect of CAD on the LAD and the slightly lower effect on the RCA. Furthermore, the investigated texture feature showed virtually no differentiating power between LAD and RCA in non-CAD patients. These findings could be further backed up by correlation plots illustrating that the correlation of the feature is lower within CAD patients compared to non-CAD patients. In summary, this indicates that plaques located exclusively in the LAD affected the PCAT of both the LAD and RCA while having the strongest effect on the PCAT of the directly underlying vessel.

Gray Level Non-Uniformity or Variability measures the variability of intensity values. In this context, a lower value reflects more homogeneity in these values. The lower values in the LAD and RCA of the CAD patients therefore suggest a more uniform PCAT texture in these patients, possibly due to global fibrosis, homogeneous inflammation, edema, or adipocyte hypertrophy. In clinical routine, the knowledge of a developing CAD before a plaque is even visible in the coronary arteries, could lead to a new way of personalized risk stratification with early treatment and hence prevention of CAD. As cardiovascular diseases are still the leading cause of death globally (1), the prevention of CAD is crucial for improving modern medicine.

Several investigations have explored the potential correlation between PCAT texture and the presence, extent, and severity of CAD. Preliminary findings suggest that specific texture patterns in PCAT are associated with vulnerable plaque characteristics, such as increased lipid content and inflammation (4, 6).

In a study conducted by Lin et al., the potential of radiomics analysis in distinguishing between patients with acute myocardial infarction (MI) and patients with stable CAD or no CAD was demonstrated. Their prospective case-control study involved the application of radiomics analysis to PCAT surrounding the proximal right coronary artery in three groups: patients with acute MI (*n* = 60), patients with stable CAD (*n* = 60), and a control group without CAD (*n* = 60). A total of 1,103 radiomics features were extracted, with 20.3% of them showing significant differences between MI patients and the control group, and 16.5% differing between MI and stable CAD patients. Building upon these findings, the study revealed that a machine-learning model that incorporated radiomics parameters along with clinical features and PCAT attenuation outperformed models that solely relied on clinical features, with or without PCAT attenuation, in detecting acute MI patients (26). This shows how the identification of texture-based biomarkers promises to offer an enhanced non-invasive means to stratify CAD risk precisely and guide clinical decision-making. Our study additionally focused on patients presenting with coronary plaques solely located in the LCA and demonstrated the existence of a more global effect on PCAT of both coronary arteries. These results could therefore facilitate the integration of radiomics into everyday clinical practice, as the analysis of any site of the PCAT could allow conclusions to be drawn about the overall risk for CAD.

In accordance with these findings, Oikonomou et al. conducted an analysis of radiomics features within PCAT in a cohort of 101 patients who experienced major adverse cardiovascular events within five years following CCTA. This analysis was compared to results from a control group of 101 patients. Once again, radiomics analysis, specifically focusing on the fat radiomics profile, which exhibited a significant increase in patients with acute MI, notably enhanced the prediction of MACE compared to traditional risk factors, including high-risk plaque features. Furthermore, their study highlighted the strong correlation between tissue inflammation and wavelet-transformed mean attenuation in adipose tissue, while fibrosis and vascularity were more closely associated with higher-order texture features (5). While our study did not primarily concentrate on overall MACE prediction but rather explored the impact of plaques in one coronary artery on the other arteries, the outcomes of the studies mentioned above emphasize the potential of radiomics analysis of PCAT. This analysis demonstrates high accuracy in risk prediction and the ability to discern various changes, such as inflammation, fibrosis, and vascularity. This suggests the potential benefits of a more targeted examination of PCAT.

Two recent studies examined the relationship between PCAT attenuation and calcified or non-calcified plaque burden specifically. In a study conducted by Giesen et al., the relationship between PCAT attenuation, CAD severity, and plaque burden in patients with chronic coronary syndrome (CCS) was investigated. This retrospective study included 868 patients undergoing CCTA for suspected or known CCS. PCAT was measured in the proximal segments of the RCA, LAD, and left circumflex artery (LCX), while CAD severity was assessed using the CAD-RADS 2.0 score. The study found significant associations between PCAT values and non-calcified plaque burden across all three coronary arteries, independent of age, the Agatston score, or the CAD-RADS 2.0 score. Specifically, PCAT attenuation was higher in patients with plaques exhibiting high-risk features (*p* < 0.05). However, only weak correlations were observed between PCAT and total plaque burden or the Agatston score, and no correlation was noted with the CAD-RADS 2.0 score (27).

Fujimoto et al. studied the relationship between calcified plaque burden (CPB), vascular inflammation, and plaque vulnerability. This study included 578 patients with coronary artery disease who underwent both CTA and optical coherence tomography (OCT). Patients were divided into four groups: one without calcification at the culprit lesion and three based on CPB tertiles. CPB was calculated as the ratio of calcified plaque volume to vessel volume at the culprit lesion. The study found that patients in the highest CPB tertile exhibited significantly lower PCAT attenuation compared to the other groups. Additionally, features of plaque vulnerability, such as lipid-rich plaques, macrophage presence, and microvessels, were least prevalent in the highest CPB tertile. Among patients with calcification, higher CPB was independently associated with older age, statin use, and lower PCAT attenuation (28).

The findings of these two studies suggest that a greater calcium burden corresponds to reduced vascular inflammation and plaque vulnerability, representing more advanced and stable plaques with diminished inflammatory activity. Higher PCAT attenuation, on the other hand, reflects localized inflammation and is closely linked to high-risk plaques and non-calcified plaques.

Studies regarding the association of a disease in one coronary artery and PCAT texture of the other coronary arteries are rare. However, a recent study by Wolny et al. described how PCAT density was higher in patients with spontaneous coronary artery dissection (SCAD) compared to the control group, while—in the SCAD group—PCAT of the SCAD-affected vessel did not significantly differ from averaged PCAT of unaffected vessels. 48 patients with SCAD were compared to 48 patients without SCAD. PCAT analysis was conducted on end-diastolic CTA reconstructions, covering the proximal 40 mm segment of all major coronary vessels, including the SCAD-related vessel. In patients with SCAD, the PCAT around the SCAD-related vessel showed no significant difference when compared to the averaged PCAT around unaffected vessels (−81.2 ± 9.2 vs. −80.6 ± 7.6, *p* = 0.74), however, when compared to patients without SCAD a significant difference was detected (−80.6 ± 7.9 vs. −85.3 ± 6.1 HU, *p* = 0.002) (29). While this study did not investigate the association of CAD and PCAT and did not focus on higher order texture features, it showcases how certain diseases in one coronary artery can be linked to structural changes in PCAT in the not directly affected coronary arteries. These results are in line with our conclusion that a CAD-affected LCA may be correlated to texture changes in PCAT of both LCA and RCA. However, our results suggest there might still be differences between PCAT of directly and indirectly affected vessels that can be uncovered with the aid of higher-order texture features.

Nonetheless, certain limitations persist within this study. Foremost, it is important to acknowledge its single-center nature and the relatively modest size of the study cohort. This study design was primarily chosen because of the novel implementation of the PCCT scanner. Consequently, a notable aspect that remains unaddressed in this study is the challenge of reproducibility associated with radiomics analysis. Standardizing texture analysis methodologies, establishing reproducibility across different PCCT platforms, and deciphering the causal relationships between texture patterns and CAD progression are crucial steps in translating our findings into clinical practice. Nevertheless, within our institution, other investigations have looked into the influence of detector type (EICT vs. PCCT) on radiomics analysis of the left ventricular myocardium, potentially offering deeper insights into texture variations through PCCT (30). The elevated stability of radiomics features observed in the context of PCCT has recently been demonstrated using a phantom model, suggesting a promising avenue for enhancing comparability through PCCT implementation (18). Nevertheless, a recent study focused on the influence of the PCCT in comparison to EICT on EAT attenuation and volume measurements. A comparison was not done in our study and should be focused on in more detail in further investigations (31). Moreover, clinical data was gathered via questionnaires and, when accessible, from medical records. As a result, some clinical data might not have been entirely captured. Especially the Body-Mass-Index could not be calculated for all patients so matching was not possible in this regard and this might have an influence on PCAT. Furthermore, even though patients without CAD were chosen to match the CAD group as ideally as possible regarding additional clinical risk factors and there was no significant difference in any of these factors, they might still have a notable impact on structural differences in PCAT texture. Additionally, this study did not take different reconstruction kernel or virtual monoenergetic images into account. Recently it has been shown that this influences PCAT (32) and should be investigated in this setting in further detail in the future. Future studies should concentrate on pericoronary adipose tissue using a prospective multicenter approach involving a more extensive study population to address these limitations.

In conclusion, PCAT texture showed structural changes in the affected LAD and non-affected RCA related to plaques exclusively located in the LCA, thereby showing that changes in PCAT texture can be detected even before plaques have formed in the underlying vessel. The investigation of pericoronary adipose tissue texture as a potential marker of CAD risk marks a significant shift in our understanding of this complex disease as it might offer the identification of future CAD in patients without plaques but suspicious PCAT. By harnessing the power of advanced imaging and computational analyses, researchers and clinicians alike will be able to gather new insights that may ultimately redefine CAD risk assessment and management—leading to a personalized risk stratification in a preventive setting.

## Data Availability

The raw data supporting the conclusions of this article will be made available by the authors, without undue reservation.
